# Chlorate of Potash in Mercurial Salivation

**Published:** 1860-05

**Authors:** 


Chlorate of Potash in Mercurial Salivation.—In a great number of cases in which the energetic use of mercurials had to be suspended, on account of salivation supervening, its continuance was rendered possible by giving to the patients, daily, from ʒ i to ʒ iss of chlorate of potash; the phenomena of mercurialization disappeared during this treatment, or very nearly so, whilst the cure of the disease was in no way retarded.— *Zeitschr. d. Wiener Aerzte.*

